# Terahertz wave modulation enhanced by laser processed PVA film on Si substrate

**DOI:** 10.1038/s41598-018-26778-7

**Published:** 2018-05-29

**Authors:** Weimin Liu, Fei Fan, Shitong Xu, Meng Chen, Xianghui Wang, Shengjiang Chang

**Affiliations:** 1 Institute of Modern Optics, Nankai University, Tianjin, 300350 China; 2 Tianjin Key Laboratory of Optoelectronic Sensor and Sensing Network Technology, Tianjin, 300350 China

## Abstract

An optically pumped ultrasensitive broadband terahertz (THz) wave modulator based on polyvinyl alcohol (PVA) film on Si wafer was demonstrated in this work. The THz time domain spectroscopy experiments confirm that the PVA/Si can drastically enhance the photo-induced THz wave modulation on the Si surface, especially when the PVA film is heated by a high-power laser. A modulation depth of 72% can be achieved only under 0.55 W/cm^2^ modulated laser power, which is superior significantly to the bare Si. The numerical simulations indicate that the laser processed PVA (LP-PVA) film increases the photo-generated carrier concentration on the Si surface in two orders of magnitude higher than that of bare Si. Moreover, the modulation mechanism and the dynamic process of laser heating on the PVA/Si have been discussed. This highly efficient THz modulation mechanism and its simple fabrication method have great application potentials in THz modulators.

## Introduction

Terahertz (THz) science and technology have been developed rapidly over the past decade due to its superiority in security, communication, and spectroscopy^1–3^. In order to manipulate THz waves efficiently, many THz functional devices have been proposed, such as THz modulators^4,5^, polarizers^6,7^, isolators^8,9^, and absorbers^10,11^. Among them, THz modulators are always a research hotspot because of its urgent demand in THz communication system. For examples, Chen *et al*. achieved an electrically controlled THz modulator based on the metamaterials in 2006, which accelerated the development of this field^12^. In 2015, Zhang *et al*. developed a metamaterial modulator with a double-channel heterostructure, initially achieving a 1 GHz modulation speed and 85% modulation depth^13^. He *et al*. proposed the graphene-SiO_2_/Si structures to achieve tunable THz resonant metamaterials with a broad tuning band and a modulation depth of 80%^14,15^. However, these modulators only work in a narrow frequency band due to the resonance properties of metamaterials.

Thus, many novel materials and broadband modulation mechanisms have been introduced to solve this problem, especially optically modulated two-dimension (2D) materials (*e.g*. graphene and MoS2)^16–21^, VO2^22,23^, doped organic film^24–30^, and nanoparticles^31,32^. For example, Weis *et al*. firstly achieved a THz modulator with a high modulation depth of 80% based on the graphene grown on the Si substrate (GOS) in 2012^18^, and Wen *et al*. developed an optically pumped THz modulator of the graphene on the Ge substrate in 2015^19^. These results show that graphene enhances the modulation depth compared with bare Si wafer under the same pump power. Fan *et al*.^20^ and Cao *et al*.^21^ found that Si-grown MoS2 has a higher modulation depth than that of GOS, and the MoS2 film induces more photo-carriers on Si surface. Moreover, similar modulation mechanism has also been found in doped organic/Si chips, for example, Yoo *et al*. reported that the photo-induced THz modulation on Si substrate can be drastically enhanced by depositing a thin organic conjugated polymer copper phthalocyanine (CuPc)^24^, and then Shen *et al*. developed several photo-induced THz modulators based on organic/Si structures, that is MEH-PPV/Si^27^, phthalocyanine compound/Si^28^, organometal halide perovskite/Si^29^, and MEH-PPV/graphene/Si^30^. These devices obtained >90% modulation depth under the pump power density of 1 W/cm^2^. These doped organic films have higher photoelectric conversion efficiency and can be more easily fabricated compared with 2D materials. However, these doped organic films are still expensive and difficult to prepare owing to their complex doped elements and organic synthesis.

In this paper, we show an optically pumped ultrasensitive broadband THz wave modulator based on a simple non-doped organic polymer polyvinyl alcohol (PVA) film on Si wafer. By experiments and numerical simulations, it is demonstrated that PVA film can drastically enhance the photo-induced THz modulation on the Si surface. Especially, after the PVA film is heated for a few minutes by a high power continuous (CW) laser, the laser processed PVA/Si (LP-PVA/Si) structure can obtain >70% modulation depth under 0.55 W/cm^2^ modulated laser. Moreover, the dynamic process of laser heating on PVA/Si and the modulation mechanism have been discussed.

## Results and Discussion

**Figure 1:**
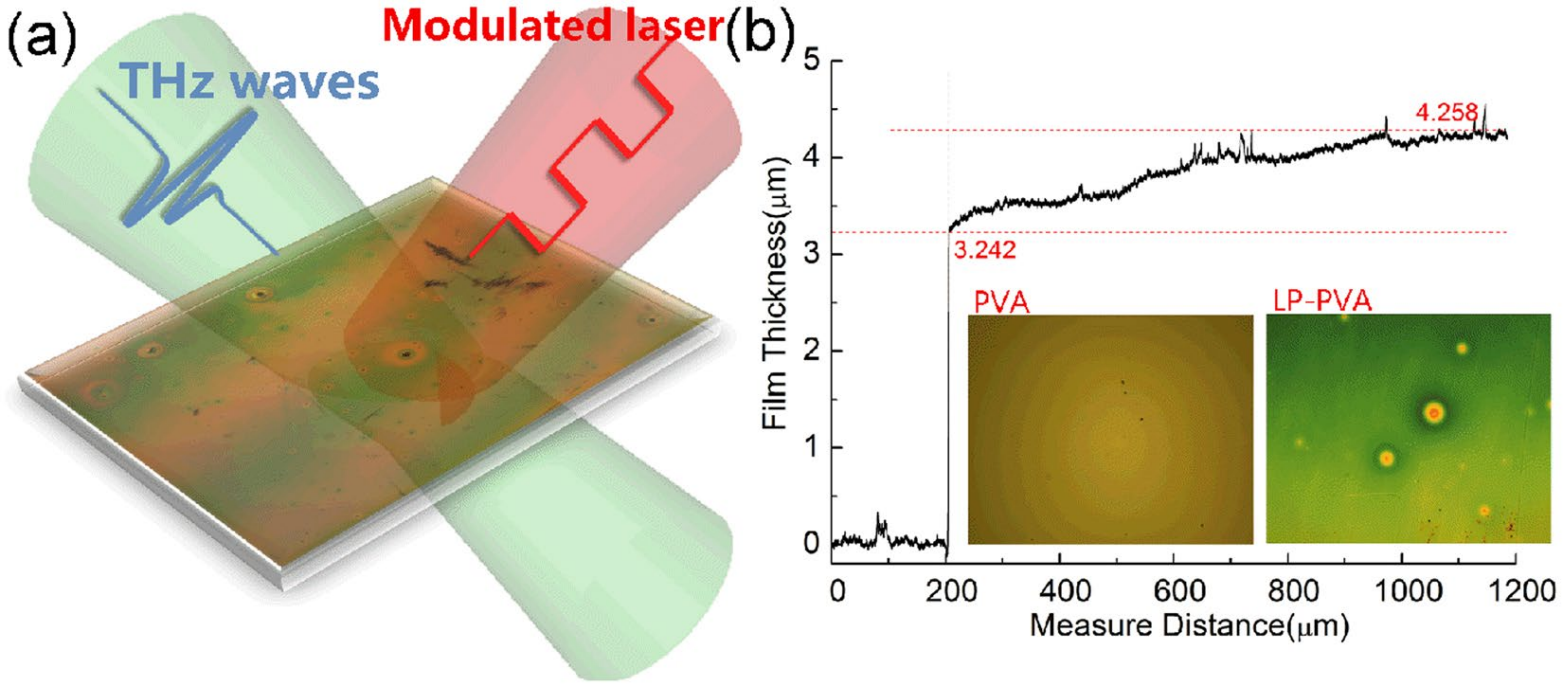
(**a**) Schematic diagram that THz waves are incident into the LP-PVA/Si pumped by a modulated laser. (**b**) LP-PVA film thickness measured by the step profiler; Insets: 10 × 50 microscope photos of PVA film without and after laser heating process.

The PVA/Si sample was prepared following the steps in Method, and the thickness of PVA film was 4.5μm accurately measured by a profilometer^33^. To obtain a LP-PVA/Si chip, the PVA/Si chip was heated by a strong 808 nm CW laser (DILAS M1F4S22-808-30C) with 7.25 W/cm^2^ of power density, and the temperature of laser spot was 220 °C measured by the infrared thermometer. After 150 s of illumination, the LP-PVA/Si chip was obtained, and its schematic diagram and micrographs are shown in Fig. 1(a,b). The thickness of PVA film without laser heating is 4.5μm uniformly, but the surface of LP-PVA/Si appears interference fringes alternated with red and green colors as shown in Fig. 1(a), and its thickness is also measured by the profilometer, as shown in Fig. 1(b).

Then, three different samples, *i.e*. the bare Si, PVA/Si, and LP-PVA/Si, were measured by THz time domain spectroscopy (THz-TDS) system^32^. THz waves are normally incident into the PVA/Si chip. We use a mechanical chopper to modulate the 808 nm CW laser to obtain a modulated laser beam, and the modulated laser beam illuminates on the PVA/Si with different pump powers. The laser spot is 0.8 cm, slightly larger than the THz spot.

**Figure 2:**
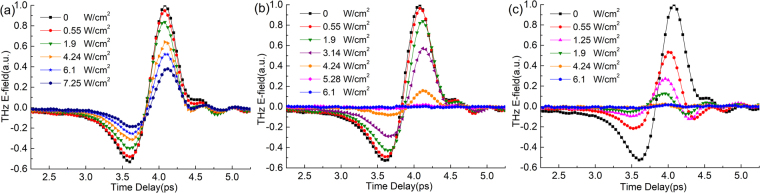
Experimentally measured THz time domain signals of (**a**) bare Si, (**b**) PVA/Si without laser heating, and (**c**) LP-PVA/Si samples under the different pump powers. The pulse peaks of three samples drop down with the increase of pump power from 0 to 7.25 W/cm^2^.

**Figure 3:**
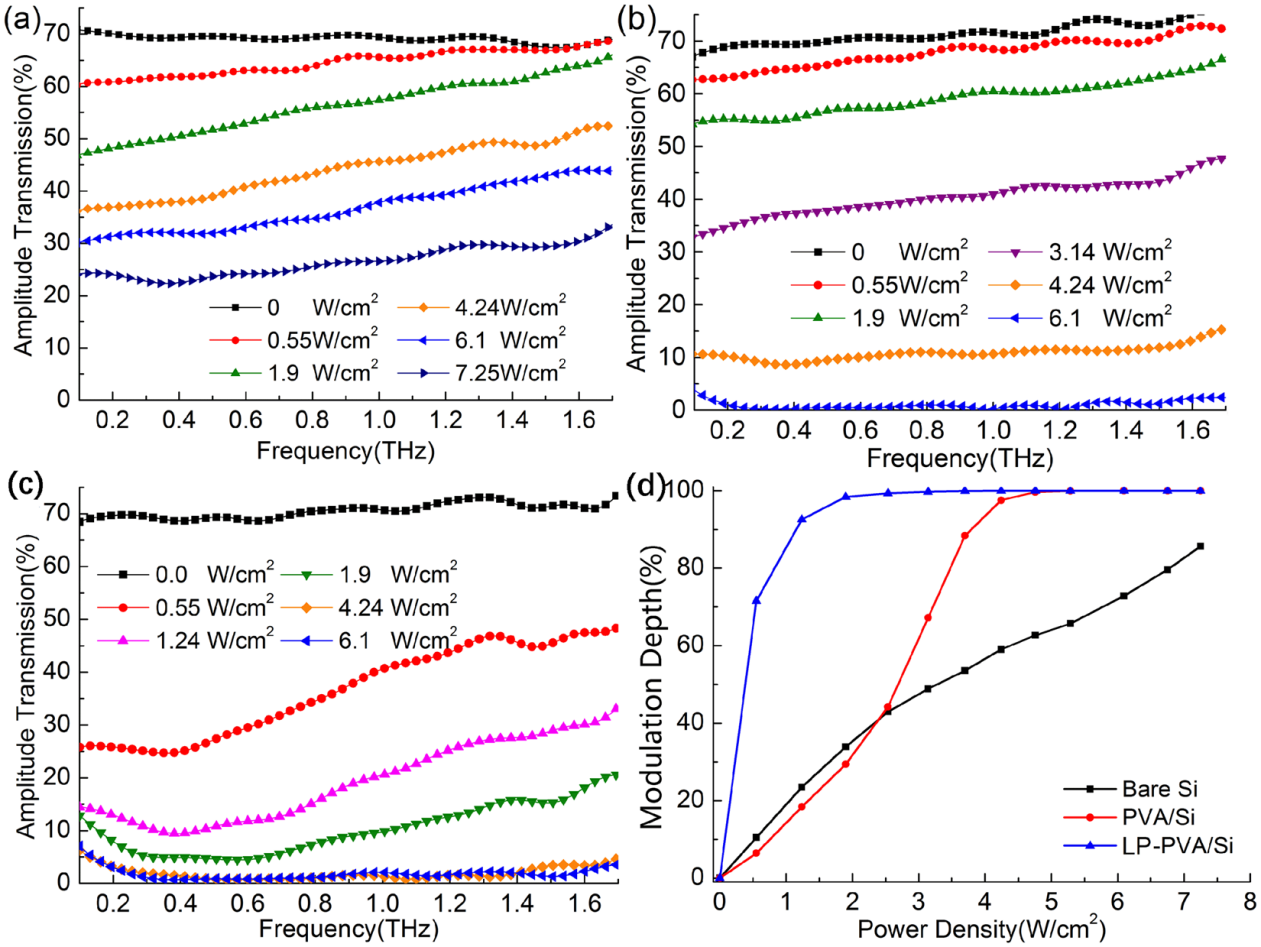
Experimental THz-TDS amplitude transmission spectra of (**a**) Bare Si, (**b**) PVA/Si without laser heating, **(c**) LP-PVA/Si samples under the different pump powers. These data are calculated from the results shown in Fig. 2. (**d**) Experimental modulation depth curves of bare Si, PVA/Si, and LP-PVA/Si change with the increase of pump power density.

The original experimental THz-TDS pulses are shown in Fig. 2, and the corresponding THz amplitude transmission spectra shown in Fig. 3(a–c) are obtained by $t(\omega )=|{E}_{s}(\omega )|/|{E}_{r}(\omega )|$, where ${E}_{r}(\omega )$ and ${E}_{s}(\omega )$ are calculated by Fourier transform of the air reference and the sample signals from Fig. 2, respectively. When the pump laser is closed, the pulse signals and transmission spectra of all three samples are nearly the same, so it is concluded that both PVA and LP-PVA films are transparent for THz waves and do not affect the transmission of Si substrate. With the increase of pump power, the pulse peaks of all three samples are all declined as shown in Fig. 2, and the low frequency components fall more than high frequency components as shown in Fig. 3. Among these three samples, the transmission of bare Si declines linearly with the increase of pump power, and PVA/Si without laser heating is similar to bare Si initially. But when the pump power density *P* > 1.9 W/cm^2^, the transmission of PVA/Si dramatically decreases. When *P* = 6.1 W/cm^2^, the amplitude transmission of PVA/Si drops down to nearly 0, while the bare Si still has 25% of amplitude transmission under 7.25 W/cm^2^, so the PVA/Si has a higher modulation depth than the bare Si when the pump power is strong. The LP-PVA/Si is quite different from the others since its transmission sharply decreases when the pump power density is very low. When *P* = 0.55 W/cm^2^, the amplitude transmission of LP-PVA/Si is only 40% at 1.0 THz; and its value drops to 0 when *P* = 4.24 W/cm^2^, much lower than 6.1 W/cm^2^ of the PVA/Si.

We also define the peak intensity modulation depth of the THz pulse, expressed as $M(P)=1-{|{E}_{p}(P)|}^{2}/{|{E}_{p}(0)|}^{2}$, where ${E}_{p}(0)$ is the peak value of the THz pulse without pump power, and ${E}_{p}(P)$ is the peak value under a pump power density *P* shown in Fig. 2. The experimental modulation depth curves of bare Si, PVA/Si, and LP-PVA/Si are shown in Fig. 3(d), which coincides with the results of transmission changes shown in Fig. 3(a–c). The modulation depth of LP-PVA/Si is dramatically increased from 72% under 0.55 W/cm^2^ to 92% under 1.2 W/cm^2^, and close to 100% under 1.9 W/cm^2^ compared to only 35% of bare Si and PVA/Si under the same pump power. For the PVA/Si, a sharp change occurs at 2.5 W/cm^2^, and it tends to be saturated at 4.25 W/cm^2^. Therefore, among these three samples, LP-PVA/Si has the highest modulation depth, and it is much more sensitive even pumped by a weak modulated laser. And the modulation property of PVA/Si without laser heating process is similar to that of bare Si pumped by the weak modulated laser, but when the modulated laser becomes stronger, the PVA/Si gets close to the LP-PVA/Si and its modulation depth is much higher than that of bare Si.

**Figure 4:**
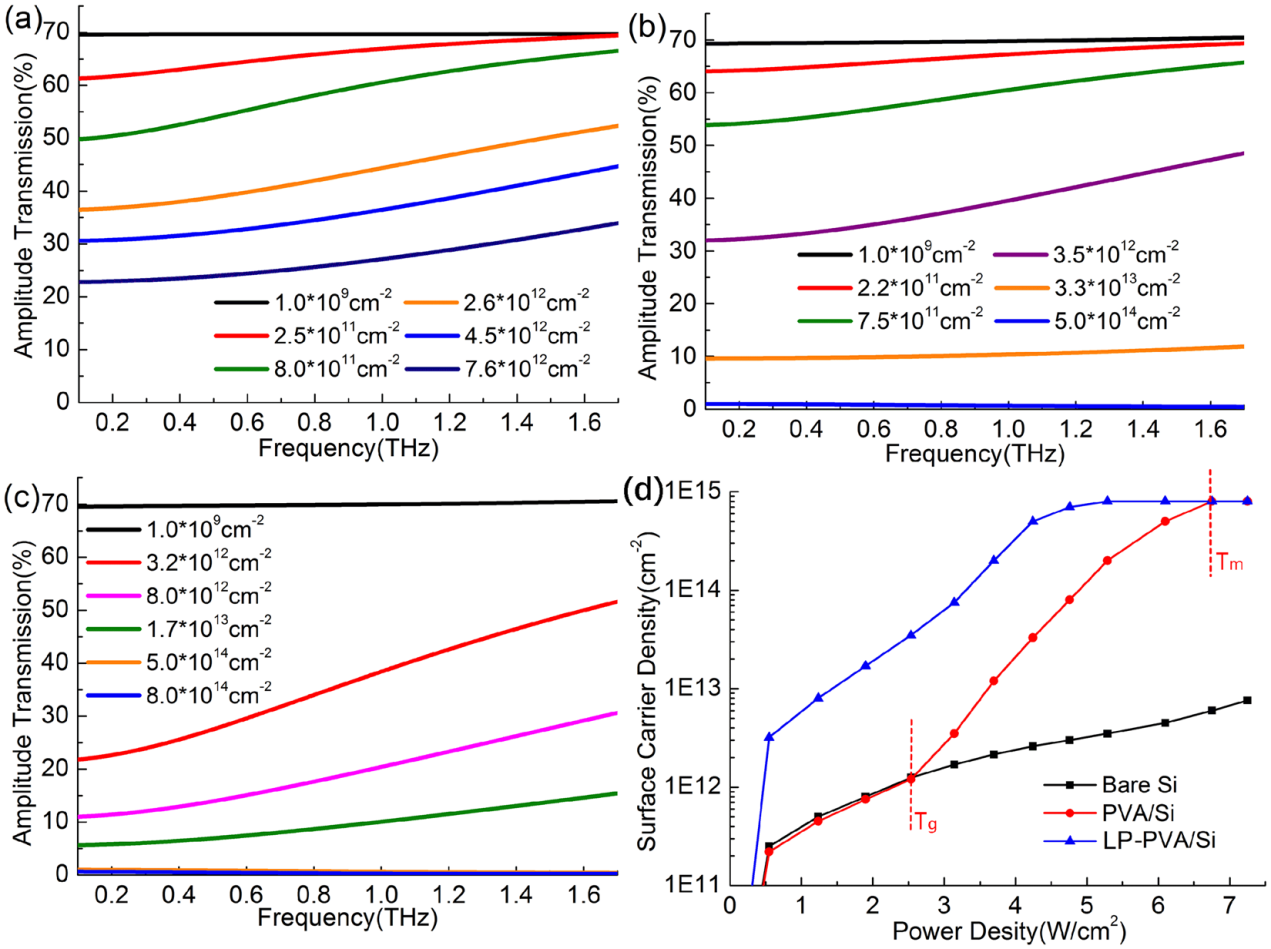
Simulative THz amplitude transmission spectra of (**a**) Bare Si, (**b)** PVA/Si, (**c**) LP-PVA/Si modeled by the different surface photon-generated carrier densities corresponding to the pump powers shown in Figs 2 and 3. (**d**) Simulative surface carrier density curves of bare Si, PVA/Si, and LP-PVA/Si change with the increase of the pump power density.

To understand the modulation mechanism of PVA/Si and LP-PVA/Si, a simulation model was built based on the photo-generated carrier absorption as Drude relaxation, to simulate the transmission spectra of three samples under the corresponding pump powers. The dielectric function of Si is expressed as $\varepsilon (\omega )={\varepsilon }_{\infty }-{\omega }_{p}^{2}/({\omega }^{2}+i\omega /\tau )$, where ${\varepsilon }_{\infty }$ = 11.7, the plasma frequency ${\omega }_{p}=\sqrt{N{e}^{2}/{\varepsilon }_{0}{m}^{\ast }}$, the relaxation time $\tau =\mu {m}^{\ast }/e$, the effective mass m^*^ = 0.98*m*_*e*_, the carrier mobility *μ* is dependent on the temperature and carrier density *N*. For example, *μ* = 1400 cm^2^V^−1^s^−1^ when *T* = 300 K and *N* = 10^15^ cm^−3^, and *μ* reduces to 100 cm^2^V^−1^s^−1^ when *N* increases to 10^19^ cm^−3 ^^34^. In the Drude model, the modulation depth drops down with the increase of the frequency. At a certain frequency, the modulation depth is mainly dependent on the photo-generated carrier density *N*, and *N* is determined by the pump power. The penetration depth of the pump laser light in the Si is non-uniform and only several hundred nanometers to 1 micrometer, so we define the surface carrier density *n*_*s*_ = *N∙d* to describe the carrier property, where *d* is the penetration depth. The Finite Difference Time Domain (FDTD) method is utilized to simulate the transmission spectra. The boundary conditions are set as the periodic boundary. The simulation structure is modeled as three layers, that is the PVA layer (4.5 μm thickness and *ε* = 2.56 without loss), active layer, and Si substrate (499 μm thickness and *ε* = 11.7 without loss). The active layer is 1μm and its *ε* is calculated according to carrier densities and their related carrier mobility *μ* as the Drude model described above. Finally, Fig. 4(a–c) is simulated to fit the spectra shown in Fig. 3(a–c), so that the relation between *n*_*s*_ and *P* can be built and shown in Fig. 4(d). It can be observed that when the pump power is weak (*P* < 2.5 W/cm^2^), the *n*_*s*_ of bare Si and PVA/Si are nearly the same, but the *n*_*s*_ of LP-PVA/Si is one order of magnitude higher than the two others. When the pump power becomes stronger (*P* > 2.5 W/cm^2^), the *n*_*s*_ of PVA/Si has a sudden increase and gradual approaches to the value of LP-PVA/Si, but the *n*_*s*_ of bare Si still increases slowly. When the pump power reaches 4.5 W/cm^2^ and 6.5 W/cm^2^, the *n*_*s*_ of LP-PVA/Si and PVA/Si respectively close to the saturated value of 8 × 10^14^ cm^−2^, which are two orders of magnitude higher than the *n*_*s*_ of bare Si under 6.5 W/cm^2^.

The reason that the PVA/Si interface produces more photo-generated carriers can be explained as follows. As shown in Fig. 4, 2.5 W/cm^2^ and 6.5 W/cm^2^ are the two critical power densities, and their corresponding temperatures of the laser spot on the PVA/Si surface are *T*_*g*_ = 80 °C and *T*_*m*_ = 200 °C, respectively. *T*_*g*_ is the glass transition temperature of PVA from the glass state to rubbery state, and *T*_*m*_ is melting point temperature from the rubbery state to molten state^35,36^. In the glass state, PVA exists as the form of long chain molecules without chain segment motion; when *T* > *T*_*g*_, the chain segment starts to vibrate and rotate; when *T* > *T*_*m*_, the whole molecule chain can move and the PVA decomposes into new polymeride with conjugate double-bonds. Importantly, the first transition is reversible when the temperature changes below *T*_*m*_, but the latter is irreversible above *T*_*m*_. Both effects induces the defect state on the Si surface, which leads more non-equilibrium carriers assembled at the PVA/Si interface, so the PVA/Si and LP-PVA/Si can greatly enhance the modulation depth for THz waves.

The above analysis can well explain the modulation property of PVA/Si without laser heating. When the low power of modulated laser is pumping on the PVA/Si, the temperature is lower than *T*_*g*_. There is no chain segment motion or conjugate double-bond in the PVA film, so the modulation behavior of the PVA/Si is the same as the bare Si. When the modulated laser becomes stronger to make *T* > *T*_*g*_, chain segment motion starts, so the PVA/Si becomes more sensitive than the bare Si under the same pump power. When the temperature of the laser spot is lower than *T*_*m*_, the PVA film can remain its original state. For example, the PVA/Si under the 6.1 W/cm^2^ modulated laser gets 100% modulation depth, but if the modulated laser power decreases to 1.9 W/cm^2^, the modulation depth will drop down to 30%. However, by a strong CW laser heating, the temperature can easily reach *T*_*m*_, so the PVA will change into the LP-PVA, but this is an irreversible process.

**Figure 5:**
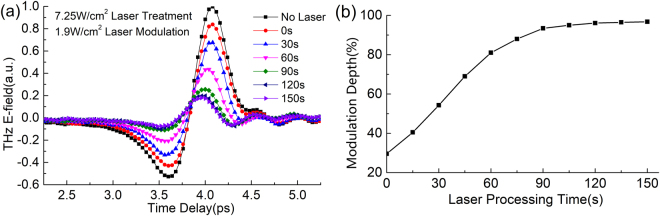
Dynamic process of the PVA/Si heated by the CW laser of 7.25 W/cm^2^ power density. (**a**) Experimental THz-TDS signals and (**b**) modulation depth curve of the PVA/Si pumped under a 1.9 W/cm^2^ modulated laser after different heating times under the 7.25 W/cm^2^ strong CW laser.

The dynamic laser heating process from PVA/Si to LP-PVA/Si is further investigated. A strong CW laser of 7.25 W/cm^2^ continuously illuminates the PVA/Si for different times, and after laser heating, we use a 1.9 W/cm^2^ modulated laser to modulate the PVA/Si and its THz-TDS signals are shown in Fig. 5(a). Figure 5(b) demonstrates that the modulation depth under 1.9 W/cm^2^ modulated laser rises up when the CW laser heating time lasts longer, which changes from the initial 30% to a saturated value of 95% after heating 120 s. Obviously, the PVA film is changed constantly due to the heating during the strong CW laser illumination. In this case, a large number of conjugated double bonds are formed, and this is an irreversible process, so after the CW laser of 7.25 W/cm^2^ heating 120 s, the modulation depth of the device under 1.9 W/cm^2^ modulated laser is 95% rather than 30%.

## Conclusions

On summary, by experiment and numerical simulation, it is confirmed that PVA/Si, especially LP-PVA/Si, can realize a highly sensitive photo-induced THz wave modulation, which enhances the photo-generated carrier concentration on the Si surface in two orders of magnitude higher than that of bare Si. A modulation depth of 72% can be achieved only under 0.55 W/cm^2^ modulated laser power, which is superior significantly to the bare Si, and this value can reach over 90% when the modulated light is larger than 1.1 W/cm^2^. Moreover, the modulation mechanism and the dynamic process of laser heating on PVA/Si have been discussed, which show that the phase transition of PVA film and the conjugate double-bonds generated during the laser heating have the critical role for photo-induced carrier enhancement in the PVA/Si structure. This non-doped organic polymer/Si chip has a good application potential in THz modulators, due to its broadband and highly efficient THz modulation mechanism and simple fabrication.

## Methods

### PVA film preparation

First, PVA solution was prepared by mixing PVA particles with de-ionized water. To accelerate the dissolution, the solution was heated to 95 °C by a water bath and stirred for hours until the dissolved PVA solution was obtained with 10 wt.%. Then the PVA film was obtained by spin coating the PVA solution on a 500 μm thickness Si wafer of 10 kΩ∙cm. Up to 10,000 r/min of the spin speed was applied to form a uniform film.

### Experiement measurement

The samples were measured by THz time domain spectroscopy (THz-TDS) system. The THz pulse was generated by a low-temperature grown GaAs photoconductive antenna (PCA) that was biased on a 100 V, 10KHz alternating signal synchronized with the phase-locked amplifier. The optical excitation source of this PCA was a Ti:sapphire laser with 75 fs duration of 80 MHz repetition rate at 800 nm. A ZnTe crystal was used for detection. All the experiments were performed at room temperature with the humidity of less than 5%. The temperatures of the laser spot on the PVA/Si surface with different pump power were measured by a non-contact infrared thermometer (UNI-T UT301C).

### Data availability

The data that support the findings of this study are available from the corresponding author on request.
